# A maternal *GOT1* novel variant associated with early-onset severe preeclampsia identified by whole-exome sequencing

**DOI:** 10.1186/s12881-020-0989-2

**Published:** 2020-03-06

**Authors:** Lin Zhang, Zheng Cao, Fan Feng, Ya-Nan Xu, Lin Li, Hong Gao

**Affiliations:** 1 Department of Internal Medicine, Beijing Obstetrics and Gynecology Hospital, Capital Medical University, Chaoyang, Beijing, 100026 China; 2 Department of Laboratory Medicine, Beijing Obstetrics and Gynecology Hospital, Capital Medical University, Chaoyang, Beijing, 100026 China; 3 Department of Basic Medical Sciences, School of Medicine, Tsinghua University, Haidian, Beijing, 100084 China; 4 Central Laboratory, Beijing Obstetrics and Gynecology Hospital, Capital Medical University, Chaoyang, Beijing, 100026 China

**Keywords:** Preeclampsia, Whole-exome sequencing, Variant, GOT1

## Abstract

**Background:**

This study wants to know the genetic cause of preeclampsia (PE) which is a leading cause of maternal and perinatal death, but the underlying molecular mechanisms that cause PE remain poorly understood. Many single nucleotide polymorphisms have been identified by genome-wide association studies and were found to be associated with PE; however, few studies have used whole-exome sequencing (WES) to identify PE variants.

**Methods:**

Five patients with severe early-onset preeclampsia (EOPE) were recruited, and WES was performed on each patient. Sanger sequencing was used to confirm the potential causative genetic variant.

**Results:**

After a stringent bioinformatics analysis, a rare variant in the *GOT1* gene, c.44C > G:p.P15R, was found in one patient. Bioinformatics analysis showed that the variant site is highly conserved across several species and was predicted to be a pathogenic variant according to several online mutational function prediction software packages. Further structural biology homology modeling suggested that P15R would change the electric environment of enzymatic center, and might affect the binding affinity of substrate or product.

**Conclusion:**

We demonstrated for the first time that the variant in *GOT1* may be associated with EOPE, the results of this study provide researchers and clinicians with a better understanding of the molecular mechanisms that underlie maternal severe EOPE.

## Background

Preeclampsia (PE) is a pregnancy-specific multi-systemic syndrome that affects several organs, including the kidneys, liver, and brain, and is a leading cause of maternal and perinatal morbidity and mortality. PE affects approximately 5% of pregnancies and is characterized by new-onset hypertension and proteinuria at ≥20 weeks of gestation [1]. The main manifestation of preeclampsia is proteinuria, as well as thrombocytopenia, impaired liver function, new development of renal insufficiency, pulmonary edema, or new-onset cerebral or visual disturbances. The placenta plays a key role in the development of this disorder. The pathogenic mechanism of PE includes defective deep placentation, oxidative and endoplasmic reticulum stress, autoantibodies to type-1 angiotensin II receptor, platelet and thrombin activation, intravascular inflammation, endothelial dysfunction, presence of an antiangiogenic state, and maternal angiogenic imbalances caused by placental antiangiogenic factors [2–8].

Genetic alteration is one of the main causes of PE [9–11], but the exact underlying molecular mechanism remains unclear. Maternal or fetal sequence variants located in or near some genes, such as *FLT1* [12, 13], *PLEKHG1* [14], *APOL1* [15], *ERAP2* [16, 17], *WNK1,* and *AKR1C3* [18], *CYP2D6* and *CYP2C9* [19], were shown to be associated with PE. Genome-wide association studies and Sanger sequencing are frequently used in the genetic analysis of PE, while high-throughput sequencing technology such as whole-exome sequencing (WES) is rarely used to find the genetic cause of PE.

Here, we recruited 5 unrelated patients with severe EOPE and performed WES for each patient. Sequence variants were filtered according to standard procedures. A novel variant in *GOT1* was identified in one patient (P1), and is considered to be a potentially pathogenic variant associated with PE, according to in silico analysis and structural biology predictions.

## Methods

### Subjects

PE can be characterized into 2 different disease entities: early-onset PE (EOPE, corresponding to preeclampsia registered at < 34 weeks) and late-onset PE (LOPE corresponding to preeclampsia registered at > 34 weeks). They are associated with different fetal and maternal effects, heritability, biochemical markers, and clinical symptoms [20]. We chose patients diagnosed with EOPE at the Beijing Obstetrics and Gynecology Hospital between January 2018 and June 2018. The diagnostic criteria of severe PE include: blood pressure of 160/110 mmHg or higher; thrombocytopenia (platelet count below 100,000/μL); impaired liver function as indicated by abnormally elevated blood concentration of liver enzymes; severe persistent right upper abdominal pain; progressive renal insufficiency (serum creatinine concentration above 1.1 mg/dL or doubling of serum creatinine concentration); pulmonary edema; and cerebral or visual disturbances. Women with high risk factors [21] for PE, such as those with chronic hypertension, pre-gestational diabetes, maternal body mass index (BMI) > 30 kg/m^2^, antiphospholipid syndrome/systemic lupus erythematosus (SLE), or who had received assisted reproduction, were excluded from our study. However, we did not rule out women with a prior history of pre-eclampsia. Using the above criteria, we identified 5 patients with severe EOPE (Table 1).

**Table 1:** Clinical features of the patients with early-onset severe preeclampsia

Variables	P1	P2	P3	P4	P5
Age (years)	35	27	29	31	31
Pro-gestational BMI (kg/m^2^)	22.9	22.1	20	26.8	22
Prior pre-eclampsia	+			+	
Registered gestational age (weeks)	28	27	27	26	31
First birth		+	+	+	+
Anemia					
Thrombocytopenia			+	+	
Impaired liver function			+	+	+
Progressive renal insufficiency	+	+	+	+	
Serous membrane fluid		+			+
Pulmonary edema			+		+
Hypoproteinemia	+		+		–
HELLP syndrome			+	+	+
Intrahepatic cholestasis of pregnancy					+
Placental abruption					
Late abortion		+	+	+	
Premature birth					+
Cesarean delivery		+	+		+
Fetal growth restriction	+				
Stillbirth	+			+	
Low birth weight infant		+	+		+

All procedures involving human participants were performed in accordance with the ethical standards of the Ethics Committee of Beijing Obstetrics and Gynecology Hospital and with the 1964 Helsinki declaration and its later amendments. Written informed consent was obtained from each patient.

### Detailed clinical information for P1

Patient No. 1 (P1) is a 35-year-old woman, body weight 62.5 kg, with a BMI of 22.9 kg/m^2^ prior to pregnancy. Six years ago, she delivered a baby girl by cesarean section because the umbilical cord was wrapped around the fetus’s neck. During the same period, she was diagnosed with severe PE (gestational age of onset is unknown), and her blood pressure returned to normal postpartum. She refused to provide a personal medical history or a family history. The patient’s blood pressure rose to 140/90 mmHg at 25 weeks of gestation, and an ultrasound examination showed that the fetus was small. She began to experience limb edema at 26 weeks of gestation but was not treated at this time. At 28 weeks of gestation, her blood pressure rose to 197/114 mmHg and urinary protein appeared as 3+, but she made no complaints. When the patient visited our hospital at that time, her body weight was 80 kg, and an ultrasound test showed that umbilical cord blood flow was S/D (the ratio of end-systolic and end-diastolic peak velocity of umbilical artery) 4.8 and fetal growth was restricted. Her urinary protein went up to 4+, and 24 h urine protein was 7431.2 mg. The highest level of serum creatine was 85.6 μmol/L, blood urea nitrogen was 10.56 mmol/L, uric acid was 472.9 mmol/L, with no detected anemia or thrombocytopenia. The lowest level of albumin was 22.6 g/L. As for liver damage, the highest level of alanine aminotransferase was 52.5 U/L, aspartate aminotransferase was 41.6 U/L, and lactate dehydrogenase was 332 U/L. The highest level of d-dimer was 10.87 mg/L. Echocardiography showed no abnormalities. Both antinuclear antibodies, antibody spectrum, and cardiolipin antibody were all negative. After administering drugs and inducing labor, P1 gave birth to a stillborn child. She recovered well and was released from the hospital 10 days later.

### Detailed clinical information for P2

Patient No. 2 (P2) is a 27-year-old woman who was gravida 1, para 0, and her BMI was 22.1 kg/m^2^ prior to pregnancy. She denied personal medical history or family history. Down screening showed high risk of open neural tube malformation at 13 weeks of gestation. The patient’s presented with high blood pressure of 178/120 mmHg during regular prenatal examination at 27 weeks of gestation, with complaint about headache, dizziness and edema of lower extremity for half a month, and nausea for a week or so. The serum creatine went up to 100.9 μmol/L within the next week. Her 24-h urine protein quantification reached 7212.5 mg/24 h. No anemia or thrombocytopenia was found. Lupus anticoagulant and anticardiolipin antibody tests were both negative. The patient received antispasmodic treatment with magnesium sulfate, and antihypertensive therapy. However, the patient developed oliguria, pleural effusion and peritoneal effusion, then she underwent cesarean section. The weight of neonatal was 885 g. Apgar score after birth was 9′-9′-9′. After the operation, the serum creatine went up to 121.2 μmol/L, with the serum urea nitrogen 11.76 mmol/L, and the uric acid 692.3 mmol/L, and the minimum level of albumin was 24.0 g/L. The patient continued to receive antihypertensive, diuretic and prophylactic anticoagulant therapy. Finally, the patient recovered well and left hospital 6 days after the operation.

### Detailed clinical information for P3

Patient No. 3 (P3) is a 29-year-old woman, who was gravida 1, para 0. Her BMI was 20.0 kg/m^2^ prior to pregnancy. She denied personal medical history or a family history. The patient’s presented with high blood pressure of 145/100 mmHg and her urinary examination showed urinary protein (3+) during regular prenatal examination at 27 weeks of gestation. She didn’t complain about headache, dizziness or edema. Her CVT indicated high peripheral resistance. Her 24-h urine protein quantification could reach 3882.0 mg/24 h. Lupus anticoagulant and anticardiolipin antibody tests were both negative. The patient received antispasmodic treatment with magnesium sulfate, and antihypertensive therapy. As a result of rapidly elevating blood pressure of 180/120 mmHg and upper abdominal pain, the patient underwent urgent cesarean section. The weight of neonatal was 1110 g. Apgar score after birth was 10′-10′-10′. One day after the operation, the platelet count fell down to 66*10^9^/L, and alanine aminotransferase (ALT) level elevated to 110.6 IU/L, aspartate aminotransferase (AST) 50.9 IU/L, lactic acid dehydrogenase (LDH) 375 IU/L, creatinine (Crea) 90.1 μmol/L, blood urea nitrogen (BUN) 9.03 mmol/L, uric acid (UA) 706.8 μmol/L. Her abdominal ultrasound showed no abnormality. She was therefore diagnosed with HELLP syndrome. The patient then received antihypertensive, diuretic and prophylactic anticoagulant therapy, and she recovered and left hospital 6 days after the operation.

### Detailed clinical information for P4

Patient No. 4 (P4) is a 31-year-old woman, who was gravida 2, para 0. Her BMI was 26.8 kg/m^2^ prior to pregnancy. And she was diagnosed with preeclampsia and intrauterine fetal death occurred at 24 weeks of gestation 3 years ago. The patient’s mother had hypertension in her old age. The patient’s presented with high blood pressure of 140/90 mmHg complaining about headache and dizziness for 3 weeks at 26 weeks of gestation. Her 24-h urine protein quantification could reach 1300 mg/24 h. Ultra-sound test showed fetal growth restriction. Umbilical cord blood flow showed B = 0, and the patient received Induction of labor by amniocentesis and rivanol. After 2 days of operation, the blood pressure rose to 190/124 mmHg, and the patient complained about upper abdominal pain. Biochemical tests showed that ALT 104.4 IU/L, AST 105.9 IU/L, UA 531.7 μmol/L, Crea 93.3 μmol/L, LDH 524 IU/L, Albumin (ALB) 31.3 g/L, routine blood test showed that her platelet account fell down to 100*10^9^/L, the haemoglobin level was in normal range. She was diagnosed with partial HELLP syndrome. Lupus anticoagulant was positive, and anticardiolipin antibody tests was negative. The patient received antihypertensive, diuretic and prophylactic anticoagulant therapy, and she recovered and left hospital 6 days after the operation.

### Detailed clinical information for P5

Patient No. 5 (P5) is a 31-year-old woman, who was gravida 1, para 0. Her BMI was 26.8 kg/m^2^ prior to pregnancy. She denied personal medical history or a family history. The patient developed pruritus at 28 weeks of gestation. She presented with high blood pressure of 170/120 mmHg with edema during regular prenatal examination at 30 weeks of gestation, and biochemical examinations showed that ALT 317.2 IU/L, AST 178.3 IU/L, total bile acide (TBA) 14.4 μmol/L, LDH 495 IU/L, Crea 92.9 μmol/L, BUN 6.84 mmol/L, UA 524.7 μmol/L, ALB 18.2 g/L, without hemolysis or thrombocytopenia. Her urinary protein was 3 + , the 24-h urine protein quantification could reach 2884.8 mg/24 h. Brain natriuretic peptide (BNP) level was 515.6 pg/ml. And ultrasound showed that massive ascites could be found. She was diagnosed with severe preeclampsia, partial HELLP syndrome and intrahepatic cholestasis of pregnancy. Then she received emergency cesarean section at 31 weeks of gestation, and the weight of the newborn baby is 1355 g. Apgar score after birth was 10′-10′-10′. After liver protecting and antihypertensive therapies, she recovered and left hospital 5 days after the operation.

### WES analysis

Genomic DNA was extracted from peripheral blood using a DNeasy Blood and Tissue Kit (Qiagen, Valencia, CA, USA). WES was performed by Annoroad Gene Technology Co., Ltd., Beijing. Briefly, exomes were captured using a SureSelect Human All Exon V6 Kit (Agilent Technologies), and were sequenced using a HiSeq X10 Sequencer (Illumina). Raw Reads were mapped against the human reference genome hg19 using Burrows-Wheeler Aligner (BWA). Single nucleotide variants (SNV) were identified by SAMTools and Genome Analysis Toolkit (GATK) software, and ANNOVAR was used for SNV functional annotation and filtering. Variants fulfilling the following criteria were retained: (i) missense, nonsense, frame-shift, or splice site variants; (ii) absent from the Exome Aggregation Consortium database (ExAC, http://exac.broadinstitute.org/), Genome Aggregation Database (gnomAD, http://gnomad.broadinstitute.org/), 1000 Genomes (1000G, http://browser.1000genomes.org/index.html), ESP6500 (http://evs.gs.washington.edu/EVS/), and our inhouse database.

### Sanger sequencing validation

The whole-exome sequencing results were validated using Sanger sequencing. For the *GOT1* (c.44C > G:p.P15R) variant, forward (5′-ATTGGTTAATCGCGTTGCCAA-3′) and reverse (5′-CCACACCTGCATCTGTAAAATGG-3′) primers were used for PCR amplification and Sanger sequencing. DNA products were electrophoresed on an ABI 3730 XL DNA sequencer (Applied Biosystems, Bedford, MA).

## Results

### WES analysis of the five patients

Five patients with severe EOPE were recruited in this study; their clinical information and data are shown in Table 1. The incidence of EOPE was about 0.5–0.8%, so the incidence of severe EOPE is much lower than EOPE. We speculated that genetic factors may play an important role in patients with severe EOPE; therefore, WES was performed for each patient. The quality of sequencing met the requirements of the bioinformatics analysis, as shown in the WES report (Supplementary Table 1).

**Fig. 1:**
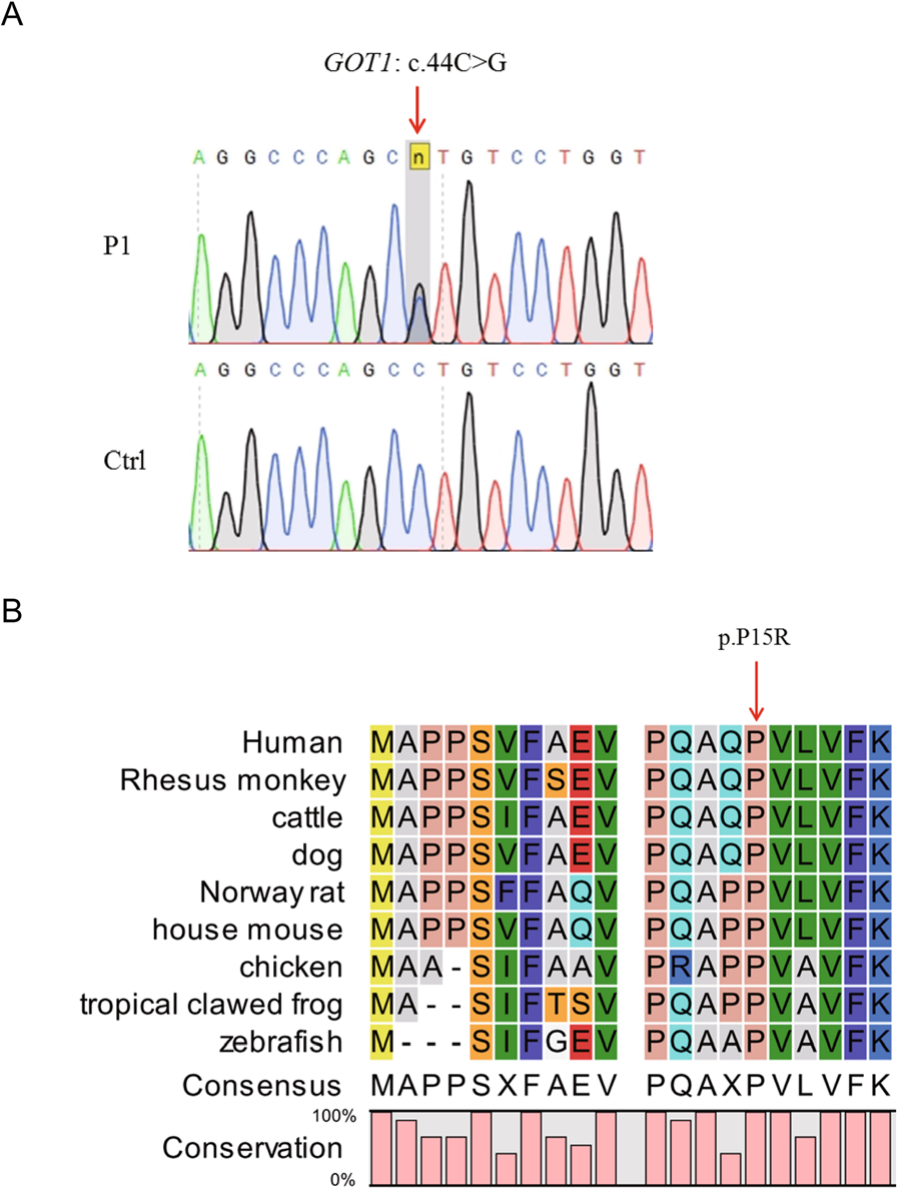
Analysis of the *GOT1* variant. **a** Sanger sequencing validated the heterozygous c.44C > G variant in the *GOT1* gene. Red arrow indicates variant site. **b** Amino acid sequence alignment of GOT1 in different species. Red arrow indicates mutated amino acid. Proline at position 15 is 100% conserved in all species

Next, we performed a filtering process on the WES data (Supplementary Table 2). First, we retained missense, nonsense, frameshift, and splice site variants, then filtered out the variants with allele frequencies above 1% in the 1000G, ESP6500, ExAC, and gnomAD databases. We then used our inhouse database to further filter out duplicate variants, and thus obtained variants for each patient. We identified 419 SNVs and 36 insertion-deletion variants (InDels) in P1, 431 SNVs and 35 InDels in P2, 449 SNVs and 38 InDels in P3, 297 SNVs and 17 InDels in P4, and 398 SNVs and 31 InDels in P5 (Supplementary Table 2). All variants were further filtered according to the list of 40 selected candidate genes that are known to be associated with severe preeclampsia [9]. In this way, we were able to narrow down the scope of the target. In the five patients, we only identified a rare variant of the *GOT1* gene c.44C > G:p.P15R in P1; the variant was confirmed by Sanger sequencing (Fig. 1a). In order to further search for genes and mutations that may be related to the occurrence of disease in other patients, we added 143 genes related to the occurrence of maternal PE to screen for mutations [9, 22, 23]. In total, we identified eight potentially pathogenic variants in P2, P3, and P4 that may be associated with PE (Supplementary Table 3). We found three variants in the *TTN* gene (Supplementary Table 3), that was consistent with the previous finding [23].

### In silico analysis of the *GOT1* variant

The Pro15 site is highly conserved across species from human to zebrafish (Fig. 1b), suggesting that Pro15 plays an important role in the function of the GOT1 protein. After searching exome and genome sequencing databases, we found that the allele frequency of c.44C > G was 0 in all databases (Table 2), indicating that the variant is extremely rare. Furthermore, several online mutational prediction tools predicted P15R to be a pathogenic variant (Table 2). We tried to use the American College of Medical Genetics and Genomics (ACMG) guidelines to classify the GOT1 variant we found. The p.P15R variant in GOT1 was classified as Uncertain Significance based on absence in large population studies (PM2), and computational predictions (PP3) according to the ACMG guidelines [24]. As the variant is heterozygous, we wanted to know whether the heterozygous variant influences disease tolerance. Constraint Metrics Z score for missense variation analysis [25] found that *GOT1* was predicted to be intolerant to variation (z = 2.17, http://exac.broadinstitute.org/gene/ENSG00000120053). Thus, the above bioinformatics analysis suggests that the rare variant c.44C > G:p.P15R of the *GOT1* gene might be potentially associated with severe EOPE.

**Table 2:** In silico analysis of *GOT1* mutation

Variants	Amino acid change	Polyphen-2^a^	SIFT^b^	PROVEAN^c^	Mutation Taster^d^	SNPs&GO^e^	FATHMM-MKL^f^	gnomAD^g^	ExAC^h^	1000 Genomes^i^	ESP6500^j^
c.44C > G	p.P15R	Possibly damaging (0.924)	Damaging (0.015)	Deleterious (−7.30)	Disease causing (0.9999)	Disease (0.918)	Damaging (0.918)	0	0	0	0

^a^Polyphen-2. Prediction Scores range from 0 to 1 with high scores indicating probably or possibly damaging

^b^SIFT, i.e., Sorting Intolerant From Tolerant. Scores vary between 0 and 1. Variants with scores close or equal to 0 are predicted to be damaging

^c^PROVEAN. Variants with scores lower than − 2.5 (cutoff) are predicted to be deleterious

^d^Mutation Taster. The probability value is the probability of the prediction, i.e., a value close to 1 indicates a high ‘security’ of the prediction

^e^SNPs&GO. Probability: Disease probability (if > 0.5 mutation is predicted Disease)

^f^FATHMM-MKL. Values above 0.5 are predicted to be deleterious, while those below 0.5 are predicted to be neutral or benign

^g^Frequency of variation in total of gnomAD database

^h^Frequency of variation in total of ExAC database

^i^Frequency of variation in 1000 Genomes database

^j^Frequency of variation in ESP6500 database

### Molecular modeling of the mutated protein

**Fig. 2:**
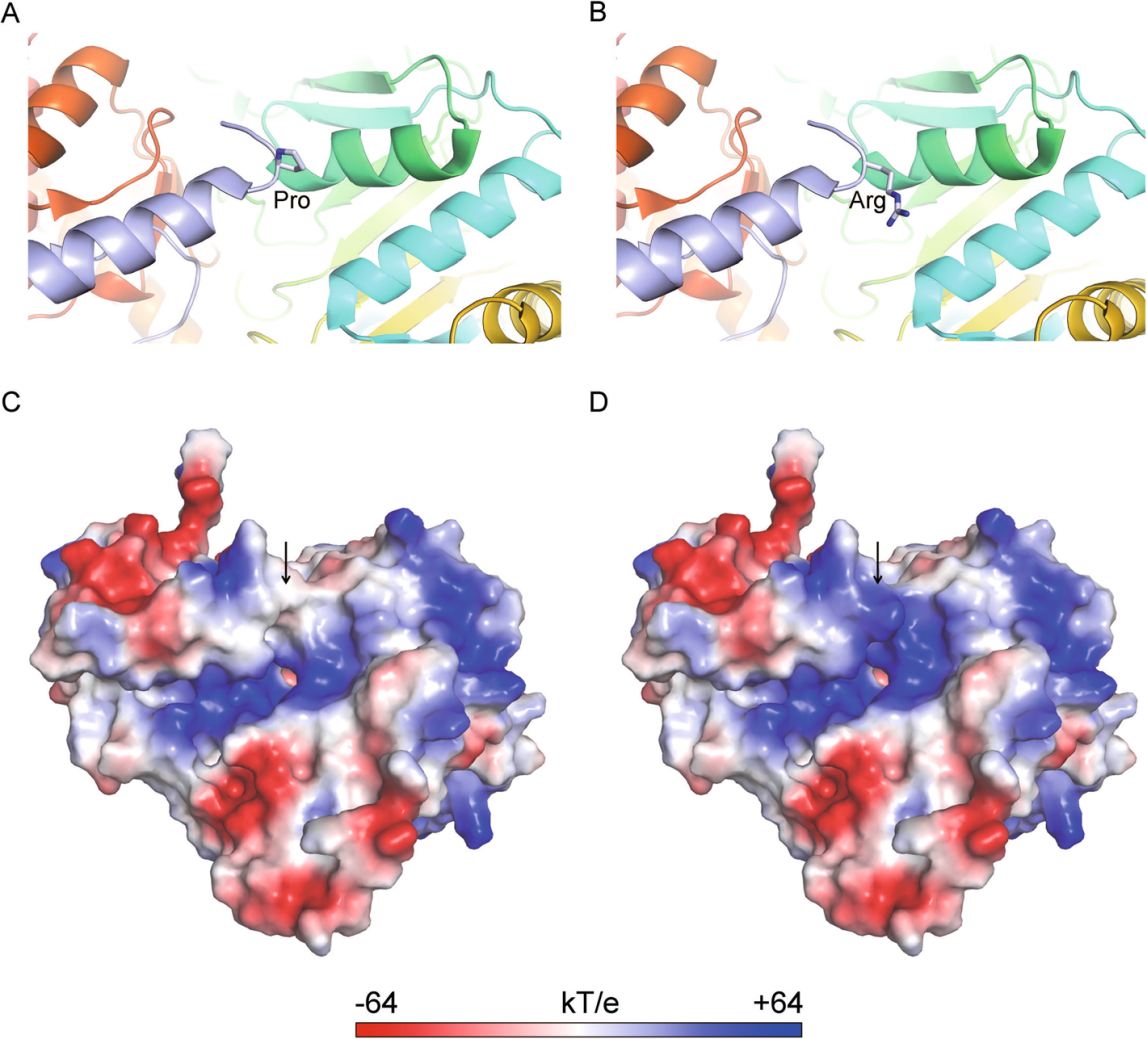
The molecular model of GOT1 protein. **a** Close view of wild type GOT1 protein structure (PDB ID: 3II0, Ugochukwu, E., Pilka, E., Cooper, C., Bray, J.E., Yue, W.W., Muniz, J., Chaikuad, A., von Delft, F., Bountra, C., Arrowsmith, C.H., Weigelt, J., Edwards, A., Kavanagh, K.L., Oppermann, U., Structural Genomics Consortium (SGC). Crystal structure of human Glutamate oxaloacetate transaminase 1 (GOT1)). The whole protein is shown as cartoon in rainbow and 15th proline side chain is shown as sticks. **b** Close view of P15R mutant type GOT1 protein structure. The whole protein is shown as cartoon in rainbow and 15th arginine side chain is shown as sticks. **c** Electrostatic surface view of the wild type GOT1 protein. Electrostatic potential is expressed as a spectrum ranging from − 64 kT/e (red) to + 64 kT/e (blue). Black arrow indicates the 15th residue proline. **d** Electrostatic surface view of the P15R variant in GOT1 protein. Electrostatic potential is expressed as a spectrum ranging from − 64 kT/e (red) to + 64 kT/e (blue). Black arrow indicates the 15th residue arginine

Fortunately, we found that the structure of GOT1 protein had been resolved (The Protein Data Bank, (PDB) 3ii0, Fig. 2a). By structure analysis, we found that there was an ‘open pocket’ at the surface of GOT1 protein, which was positive charged (Fig. 2c). The 15th residue proline was located at the edge of this pocket (Fig. 2c). In our study, this residue mutated to arginine (Fig. 2b). Arginine is a basic amino acid with a big side chain (Fig. 2d). We proposed that this point mutation would change the electric environment of enzymatic center, meanwhile, affect the binding affinity of substrate or product even more the whole process of enzyme-catalyzed reactions. Computer modeling had confirmed our proposal that P15R variant changed the electricity and enhanced the positive charge of this area (Fig. 2d).

## Discussion

In this study, whole-exome sequencing was performed in 5 patients with severe EOPE. A rare variant in the *GOT1* gene, c.44C > G:p.P15R was found in one patient (P1). Bioinformatics analysis showed that the variant was highly conserved across different species and was predicted to be a pathogenic variant according to several online mutational function prediction software packages. Further structural biology homology modeling suggested that P15R may change the electricity and enhance the positive charge of this area.

The *GOT1* gene—full name glutamate oxaloacetate transaminase—is also known as aspartate aminotransferase. GOT1 can catalyze L-cysteine or L-aspartate substrates. L-cysteine can be converted into 3-mercaptopyruvate by GOT1, and then H_2_S can be obtained by 3-mercaptopyruvate sulphurtransferase. L-aspartate can be catalyzed into L-glutamate by GOT1, which is a pyridoxal phosphate dependent enzyme found in the cytoplasm.

Previous studies suggest that maternal aspartate metabolic or catalytic processes are associated with severe preeclampsia [9]. A study using high-resolution magic angle spinning nuclear magnetic resonance spectroscopy (HR-MAS MRS) found that aspartate levels are higher in the placenta of patients with preeclampsia, while glutamate levels are lower [26]. Another study using ultra performance liquid chromatography-mass spectrometry (UPLC-MS) found that glutamate levels were low in explanted placental villous fragment cultures of preeclampsia patients [27]. The P15R variant identified in the present study is located near the GOT1 enzyme activity center. Arginine is an alkaline amino acid, while both the substrate (L-aspartate) and product (L-glutamate) involved in GOT1 reaction are acidic. The mutated protein thus, may enhance alkalinity near the active center of the enzyme, which may increase the binding ability of the substrate or the product, and in turn affect the release of the product. Thus, the decrease in L-glutamate release due to the P15R variant in the *GOT1* gene could be related to the occurrence of preeclampsia in this patient.

A previous study found that hydrogen sulfide (H_2_S) is essential for a healthy placental vasculature, and that a decrease in H_2_S activity is related to the onset of preeclampsia [28]. H_2_S can cause contraction and relaxation of human umbilical vein endothelial cells [29]. The formation of hydrogen sulfide is catalyzed by GOT1, which is expressed by human umbilical artery endothelial and smooth muscle cells [29]. Thus, the P15R variant may reduce the release of 3-mercaptopyruvate, thereby affecting the production of hydrogen sulfide, which could then induce preeclampsia owing to an abnormal placental vasculature.

Previous epidemiological studies have suggested that genetic factors account for about 55–60% of PE cases, of which 30–35% can be attributed to maternal genetic factors and 20% to fetal genetic factors [11, 30, 31]. Therefore, one third of PE may be related to maternal genetic factors. In this study, we tried to find potential genetic pathogenic factors in 5 patients with severe EOPE, and the range of genetic genes was based on 40 gene panels related to severe EOPE. Only one patient with severe EOPE was found to have a potential pathogenic genetic variant, and no other four patients were found to have a candidate pathogenic variant. Before the study, we thought that we might find a higher proportion of genetic variants in EOPE. But at present, only 20% (one patient) of the genetic pathogenicity was found, which may be related to the small number of genes in the gene panel we used. We speculated that there may be more than 40 genes that can lead to severe EOPE. In the future, with the further study of PE genetics, the gene list will be more perfect, and more cases that we can find the cause will be found.

## Conclusion

In summary, the P15R variant in the *GOT1* gene identified in the present study may lead to preeclampsia by causing abnormal synthesis of glutamate or hydrogen sulfide. Furthermore, the findings suggest that abnormal aspartate metabolic or catalytic processes, together with placental vasculature contraction or relaxation abnormalities, may be associated with preeclampsia. This study provides a genetic basis for the etiology and molecular mechanisms that underlie preeclampsia.

## Supplementary information

**Additional file 1: Supplementary Table 1.** Statistics of the aligned data. **Supplementary Table 2.** Outline of filtering the polymorphisms. **Supplementary Table 3.** Sequence variants identified that is associated with preeclampsia.

## Data Availability

The datasets used and/or analysed during the current study have been deposited in the European Variation Archive (EVA) under the following accessions: https://www.ebi.ac.uk/ena/data/view/PRJEB36886.

## Abbreviations

1000G: 1000 Genomes
ACMG: American College of Medical Genetics and Genomics
ALB: Albumin
ALT: Alanine aminotransferase
AST: Aspartate aminotransferase
BMI: Body mass index
BNP: Brain natriuretic peptide
BUN: Blood urea nitrogen
BWA: Burrows-Wheeler Aligner
Crea: Creatinine
EOPE: Early-onset preeclampsia
ExAC: Exome Aggregation Consortium
GATK: Genome Analysis Toolkit
gnomAD: Genome Aggregation Database
H_2_S: Hydrogen sulfide
HR-MAS MRS: High-resolution magic angle spinning nuclear magnetic resonance spectroscopy
InDels: Insertion-deletion variants
LDH: Lactic acid dehydrogenase
LOPE: Late-onset preeclampsia
P1: Patient No. 1
PDB: The Protein Data Bank
PE: Preeclampsia
S/D: The ratio of end-systolic and end-diastolic peak velocity of umbilical artery
SLE: Systemic lupus erythematosus
SNV: single nucleotide variants
TBA: Total bile acide
UA: Uric acid
UPLC-MS: Ultra performance liquid chromatography-mass spectrometry
WES: Whole-exome sequencing

## References

[1] Zhang J, Meikle S, Trumble A (2003). Severe maternal morbidity associated with hypertensive disorders in pregnancy in the United States. Hypertens Pregnancy.

[2] Roberts JM (2000). Preeclampsia: what we know and what we do not know. Semin Perinatol.

[3] Roberts JM, Taylor RN, Musci TJ, Rodgers GM, Hubel CA, McLaughlin MK (1989). Preeclampsia: an endothelial cell disorder. Am J Obstet Gynecol.

[4] Stillman IE, Karumanchi SA (2007). The glomerular injury of preeclampsia. J Am Soc Nephrol.

[5] Roberts JM (1998). Endothelial dysfunction in preeclampsia. Semin Reprod Endocrinol.

[6] Redman CW, Sargent IL (2003). Pre-eclampsia, the placenta and the maternal systemic inflammatory response--a review. Placenta.

[7] Craici IM, Wagner SJ, Weissgerber TL, Grande JP, Garovic VD (2014). Advances in the pathophysiology of pre-eclampsia and related podocyte injury. Kidney Int.

[8] Powers RW, Roberts JM, Plymire DA, Pucci D, Datwyler SA, Laird DM (2012). Low placental growth factor across pregnancy identifies a subset of women with preterm preeclampsia: type 1 versus type 2 preeclampsia?. Hypertension.

[9] Triche EW, Uzun A, DeWan AT, Kurihara I, Liu J, Occhiogrosso R (2014). Bioinformatic approach to the genetics of preeclampsia. Obstet Gynecol.

[10] Mogren I, Hogberg U, Winkvist A, Stenlund H (1999). Familial occurrence of preeclampsia. Epidemiology.

[11] Nilsson E, Salonen Ros H, Cnattingius S, Lichtenstein P (2004). The importance of genetic and environmental effects for pre-eclampsia and gestational hypertension: a family study. BJOG.

[12] McGinnis R, Steinthorsdottir V, Williams NO, Thorleifsson G, Shooter S, Hjartardottir S (2017). Variants in the fetal genome near FLT1 are associated with risk of preeclampsia. Nat Genet.

[13] Gray KJ, Saxena R, Karumanchi SA (2018). Genetic predisposition to preeclampsia is conferred by fetal DNA variants near FLT1, a gene involved in the regulation of angiogenesis. Am J Obstet Gynecol.

[14] Gray KJ, Kovacheva VP, Mirzakhani H, Bjonnes AC, Almoguera B, DeWan AT (2018). Gene-centric analysis of preeclampsia identifies maternal association at PLEKHG1. Hypertension.

[15] Reidy KJ, Hjorten RC, Simpson CL, Rosenberg AZ, Rosenblum SD, Kovesdy CP (2018). Fetal-not maternal-APOL1 genotype associated with risk for preeclampsia in those with African ancestry. Am J Hum Genet.

[16] Hill LD, Hilliard DD, York TP, Srinivas S, Kusanovic JP, Gomez R (2011). Fetal ERAP2 variation is associated with preeclampsia in African Americans in a case-control study. BMC Med Genet.

[17] Johnson MP, Roten LT, Dyer TD, East CE, Forsmo S, Blangero J (2009). The ERAP2 gene is associated with preeclampsia in Australian and Norwegian populations. Hum Genet.

[18] Sun CJ, Li L, Li X, Zhang WY, Liu XW (2018). Novel SNPs of WNK1 and AKR1C3 are associated with preeclampsia. Gene.

[19] Sun CJ, Li L, Li XY, Zhang WY, Liu XW (2018). Associations of polymorphisms of CYP2D6 and CYP2C9 with early onset severe pre-eclampsia and response to labetalol therapy. Arch Gynecol Obstet.

[20] Raymond D, Peterson E (2011). A critical review of early-onset and late-onset preeclampsia. Obstet Gynecol Surv.

[21] Bartsch E, Medcalf KE, Park AL, Ray JG (2016). Clinical risk factors for pre-eclampsia determined in early pregnancy: systematic review and meta-analysis of large cohort studies. BMJ.

[22] Melton PE, Johnson MP, Gokhale-Agashe D, Rea AJ, Ariff A, Cadby G (2019). Whole-exome sequencing in multiplex preeclampsia families identifies novel candidate susceptibility genes. J Hypertens.

[23] Gammill HS, Chettier R, Brewer A, Roberts JM, Shree R, Tsigas E (2018). Cardiomyopathy and preeclampsia. Circulation.

[24] Richards S, Aziz N, Bale S, Bick D, Das S, Gastier-Foster J (2015). Standards and guidelines for the interpretation of sequence variants: a joint consensus recommendation of the American College of Medical Genetics and Genomics and the Association for Molecular Pathology. Genet Med.

[25] Samocha KE, Robinson EB, Sanders SJ, Stevens C, Sabo A, McGrath LM (2014). A framework for the interpretation of de novo mutation in human disease. Nat Genet.

[26] Austdal M, Thomsen LC, Tangeras LH, Skei B, Mathew S, Bjorge L (2015). Metabolic profiles of placenta in preeclampsia using HR-MAS MRS metabolomics. Placenta.

[27] Dunn WB, Brown M, Worton SA, Crocker IP, Broadhurst D, Horgan R (2009). Changes in the metabolic footprint of placental explant-conditioned culture medium identifies metabolic disturbances related to hypoxia and pre-eclampsia. Placenta.

[28] Wang K, Ahmad S, Cai M, Rennie J, Fujisawa T, Crispi F (2013). Dysregulation of hydrogen sulfide producing enzyme cystathionine gamma-lyase contributes to maternal hypertension and placental abnormalities in preeclampsia. Circulation.

[29] Mohammed R, Provitera L, Cavallaro G, Lattuada D, Ercoli G, Mosca F (2017). Vasomotor effects of hydrogen sulfide in human umbilical vessels. J Physiol Pharmacol.

[30] Cnattingius S, Reilly M, Pawitan Y, Lichtenstein P (2004). Maternal and fetal genetic factors account for most of familial aggregation of preeclampsia: a population-based Swedish cohort study. Am J Med Genet A.

[31] Esplin MS, Fausett MB, Fraser A, Kerber R, Mineau G, Carrillo J (2001). Paternal and maternal components of the predisposition to preeclampsia. N Engl J Med.

