# Supplementary material for: A maternal GOT1 novel variant associated with early-onset severe preeclampsia identified by whole-exome sequencing
Source: BMC Med Genet. 2020 Mar 6;21:49. doi: 10.1186/s12881-020-0989-2 (PMC7060644; doi:10.1186/s12881-020-0989-2)
Supplement: Supplementary file 1 — Additional file 1: Supplementary Table 1. Statistics of the aligned data. Supplementary Table 2. Outline of filtering the polymorphisms. Supplementary Table 3. Sequence variants identified that is associated with preeclampsia. [file 12881_2020_989_MOESM1_ESM.docx]

**Supplementary Table 1.** Statistics of the aligned data

| **Samples** | **P1** | **P2** | **P3** | **P4** | **P5** |
| --- | --- | --- | --- | --- | --- |
| Target Region (bp) | 60,456,963 | 60,456,963 | 60,456,963 | 60,268,283 | 60,456,963 |
| Clean Reads | 78,452,810 | 99,872,934 | 98,765,276 | 90,160,472 | 89,595,150 |
| Clean Bases | 11,767,921,500 | 14,980,940,100 | 14,814,791,400 | 13,524,070,800 | 13,439,272,500 |
| Mapped Reads | 78,360,413 | 99,723,778 | 98,636,434 | 90,081,624 | 89,502,164 |
| Mapped Bases | 11,691,108,203 | 14,860,087,969 | 14,710,808,676 | 13,416,855,088 | 13,358,411,510 |
| Mapping Rate (%) | 99.88 | 99.85 | 99.87 | 99.91 | 99.9 |
| Reads Mapped to Target Region | 59,939,846 | 55,721,591 | 70,047,464 | 69,578,484 | 69,619,694 |
| Capture Specificity (%) | 76.49 | 55.88 | 71.02 | 77.24 | 77.79 |
| Duplication Rate (%) | 14.49 | 13.38 | 14.27 | 27.13 | 15.52 |
| Uniq Rate(%) | 96.1 | 96.29 | 96.1 | 96.86 | 96.03 |
| Bases Mapped to Target Region | 7,099,121,411 | 6,570,010,751 | 8,284,498,769 | 8,338,800,550 | 8,262,434,526 |
| Mean Depth of Target Region | 117.42 | 108.67 | 137.03 | 138.36 | 136.67 |
| Coverage of Target Region (%) | 99.46 | 99.42 | 99.46 | 99.8 | 99.34 |
| Fraction of Target Covered >=4X | 99.13 | 99.04 | 99.16 | 99.66 | 99.01 |
| Fraction of Target Covered >=10X | 98.47 | 98.3 | 98.61 | 99.34 | 98.4 |
| Fraction of Target Covered >=20X | 97.16 | 96.81 | 97.57 | 98.55 | 97.32 |
| Fraction of Target Covered >=30X | 95.33 | 94.76 | 96.23 | 97.21 | 95.93 |
| Fraction of Target Covered >=50X | 88.6 | 87.18 | 91.84 | 91.6 | 91.33 |
| Bases Mapped to Flanking Region | 2,892,695,743 | 2,724,856,654 | 3,394,629,606 | 3,208,131,250 | 3,340,277,212 |
| Mean Depth of Flanking Region | 61.59 | 58.01 | 72.27 | 68.55 | 71.12 |
| Coverage of Flanking Region (%) | 95.72 | 95.7 | 95.75 | 96.16 | 95.58 |
| Fraction of Flanking Covered >=4X | 94.97 | 94.93 | 95.13 | 95.57 | 94.9 |

**Supplementary Table 2.** Outline of filtering the polymorphisms

| **Samples** | P1 | P2 | P3 | P4 | P5 |
| --- | --- | --- | --- | --- | --- |
| Exon Enrichment kit | SureSelect Human All Exon V6 | | | | |
| Platform | Hiseq PE150 150 bp | | | | |
| **Total variation** |  |  |  |  |  |
| Single nucleotide variants | 97,838 | 97,501 | 98,196 | 81,003 | 97,999 |
| Indels | 14,967 | 14,973 | 15,350 | 10,428 | 15,229 |
| **Missense, nonsense, frameshift and splice site variants** |  |  |  |  |  |
| Single nucleotide variants | 11,233 | 10,917 | 11,179 | 9,364 | 11,034 |
| Indels | 636 | 629 | 647 | 578 | 642 |
| **Public databases MAF < 1%** |  |  |  |  |  |
| Single nucleotide variants | 613 | 585 | 621 | 476 | 563 |
| Indels | 96 | 75 | 80 | 71 | 77 |
| **Variants were further filtered**  **by checking our inhouse databases** |  |  |  |  |  |
| Single nucleotide variants | 419 | 431 | 449 | 297 | 398 |
| Indels | 36 | 35 | 38 | 17 | 31 |
|  |  |  |  |  |  |

MAF, minor allele frequency.

**Supplementary Table 3.** Sequence variants identified that is associated with preeclampsia.

| Patient ID | Gene | Nucleotide change | Amino acid change | Polyphen2 prediction | SIFT prediction |
| --- | --- | --- | --- | --- | --- |
| P2 | *FKTN* | c.A1027C | p.K343Q | Probably damaging | Damaging |
| P2 | *MYH7* | c.G3803A | p.R1268H | Probably damaging | Damaging |
| P2 | *TTN* | c.C49544T | p.T16515M | Probably damaging | Damaging |
| P2 | *TTN* | c.C53359T | p.R17787C | Probably damaging | Damaging |
| P3 | *COL18A1* | c.C1108G | p.R370G | Probably damaging | Damaging |
| P3 | *SYNE1* | c.T10616G | p.L3539W | Probably damaging | Damaging |
| P4 | *CTLA4* | c.C505G | p.P169A | Possibly damaging | Damaging |
| P4 | *TTN* | c.C28730T | p.P9577L | Probably damaging | Damaging |
